# Robotische Hernienchirurgie II: Robotische primär ventrale und inzisionale Hernienversorgung (rv-TAPP und r-Rives/r-TARUP). Videobeitrag und Ergebnisse einer Kohortenstudie an 118 Patienten

**DOI:** 10.1007/s00104-021-01450-5

**Published:** 2021-07-13

**Authors:** Johannes Baur, Michaela Ramser, Nicola Keller, Filip Muysoms, Jörg Dörfer, Armin Wiegering, Lukas Eisner, Ulrich A. Dietz

**Affiliations:** 1 Klinik für Viszeral‑, Gefäss- und Thoraxchirurgie, Kantonsspital Olten, Baslerstrasse 150, 4600 Olten, Schweiz; 2 Klinik für Allgemein‑, Viszeral- und Gefässchirurgie, Kantonsspital Baden, Im Engel 1, 5404 Baden, Schweiz; 3 Department of Surgery, AZ Maria Middelares, Buitenring Sint-Denijs 30, 9000 Gent, Belgien; 4 Klinik und Poliklinik für Allgemein‑, Viszeral‑, Transplantations‑, Gefäß- und Kinderchirurgie, Universitätsklinikum Würzburg, Oberdürrbacher Straße 6, 97080 Würzburg, Deutschland

**Keywords:** Umbilikalhernie, Inzisionalhernie, Primär ventrale Hernie, Minimalinvasiv, Retrorektus Netz, Linea alba, Umbilical hernia, Incisional hernia, Primary ventral hernia, Minimally invasive, Retrorectus mesh, Linea alba

## Abstract

Die endoskopische Versorgung von Umbilikal- und Inzisionalhernien hat sich in den vergangenen 30 Jahren an die Limitationen der konventionellen laparoskopischen Instrumente angepasst. Dazu gehört die Entwicklung von Netzen für die intraperitoneale Lage (intraperitoneales Onlay-Mesh, IPOM) mit antiadhäsiven Beschichtungen; allerdings kommt es bei einem beträchtlichen Teil dieser Patienten doch zu Adhäsionen. Minimal-invasive Verfahren führen zu weniger perioperativen Komplikationen, bei einer etwas höheren Rezidivrate. Mit den ergonomischen Ressourcen der Robotik, die abgewinkelte Instrumente anbietet, besteht erstmals die Möglichkeit, Netze minimal-invasiv in unterschiedliche Bauchdeckenschichten zu implantieren und gleichzeitig eine morphologische und funktionelle Rekonstruktion der Bauchdecke zu erreichen. In diesem Videobeitrag wird die Versorgung von Ventral- und Inzisionalhernien mit Netzimplantation in den präperitonealen Raum (robotische ventrale transabdominelle präperitoneale Patchplastik, rv-TAPP) sowie in den retrorektalen Raum (r-Rives bzw. robotische transabdominelle retromuskuläre umbilikale Patchplastik [r-TARUP]) präsentiert. Es werden die Ergebnisse einer Kohortenstudie an 118 konsekutiven Patienten vorgestellt und im Hinblick auf den Mehrwert der robotischen Technik in der Extraperitonealisierung der Netze und in der Weiterbildung diskutiert.

**Video online:**

Die Onlineversion dieses Beitrags (10.1007/s00104-021-01450-5) enthält drei Videos und ein Merkblatt/OP-Check-Liste (Supplement Material 1).

## Abstract (en)

Endoscopic management of umbilical and incisional hernias has adapted to the limitations of conventional laparoscopic instruments over the past 30 years. This includes the development of meshes for intraperitoneal placement (intraperitoneal onlay mesh, IPOM), with antiadhesive coatings; however, adhesions do occur in a significant proportion of these patients. Minimally invasive procedures result in fewer perioperative complications, but with a slightly higher recurrence rate. With the ergonomic resources of robotics, which offers angled instruments, it is now possible to implant meshes in a minimally invasively manner in different abdominal wall layers while achieving morphologic and functional reconstruction of the abdominal wall. This video article presents the treatment of ventral and incisional hernias with mesh implantation into the preperitoneal space (robot-assisted transabdominal preperitoneal ventral hernia repair, r‑ventral TAPP) as well as into the retrorectus space (r-Rives and robotic transabdominal retromuscular umbilical prosthetic repair, r‑TARUP, respectively). The results of a cohort study of 118 consecutive patients are presented and discussed with regard to the added value of the robotic technique in extraperitoneal mesh implantation and in the training of residents.

## Hintergrund

Auch wenn primär ventrale Hernien und inzisionale Hernien unterschiedliche Entitäten mit entsprechend unterschiedlichen Indikationen, perioperativen Herausforderungen und nicht vergleichbarer Rezidivprognose sind, werden doch beide Entitäten bis zu einem gewissen Grad chirurgisch ähnlich versorgt. Wichtig ist bei der Auswertung, dass beide Subgruppen gesondert analysiert werden.

Es gibt eine große Vielfalt an Operationsverfahren:

- der periumbilikale Zugang mit präperitonealer Netzeinlage (präperitoneale umbilikale Mesh-Plastik, PUMP; [1]),
- offene Zugänge für die Retrorektus- oder IPOM(intraperitoneales Onlay-Mesh)-Position und
- minimal-invasive Verfahren, entweder laparoskopisch (IPOM, mit oder ohne Bruchlückenverschluss) oder transumbilikal als E/MILOS („endoscopic/mini to less open sublay“; [2, 3].

Patienten höheren Alters und mit höherem Body-Mass-Index (BMI) profitieren am meisten von minimal-invasiven Verfahren, da das Komplikationsrisiko geringer ist. Die Daten zeigen allerdings, dass die Rezidivrate beim laparoskopischen IPOM größer ist als bei offenen morphologischen und funktionellen Rekonstruktionen [2]. Bei IPOM-Netzen kommt es zusätzlich zu den postoperativen Schmerzen immer wieder zu Adhäsionen zum Darm und in seltenen Fällen zu Darmarrosionen [4]. In Anlehnung an den ersten Satz in Patricia Highsmiths Roman „Der amerikanische Freund“ (1974) kann postuliert werden, dass es die perfekte Operation nicht gibt, denn solange es Mensch und Wissenschaft geben wird, wird es auch Idiosynkrasien und neue Verfahren geben.

Die Robotik eröffnet neue Wege: Sie ermöglicht im Vergleich zur konventionellen Laparoskopie den technisch einfachen Zugang zu den unterschiedlichen Schichten der Bauchdecke und integriert die Vorteile offener Verfahren (weniger Rezidive) mit denen minimal-invasiver Verfahren (weniger Komplikationen); dabei können einerseits die Schichten morphologisch und funktionell rekonstruiert, andererseits ausreichend große Netze implantiert werden. Die Robotik hat im Arbeitsablauf der Operation merkbare Vorteile gegenüber konventionellen minimal-invasiven Verfahren: Ergonomie, Freiheitsgrade der Instrumente, Bildstabilität und Immersionsblick sind nur einige davon. Bei Bedarf kann bei kardiopulmonal belasteten Patienten mit einem niedrigeren intraperitonealen Druck gearbeitet werden, da die Ports (wie in der Lift-Laparoskopie) die Bauchdecke halten.

In diesem Beitrag werden die Ergebnisse einer Kohorte mit robotisch durchgeführter präperitonealer (robotische ventrale transabdominelle präperitoneale Patchplastik, rv-TAPP) und retrorektaler Netzpositionierung (r-Rives bzw. robotische transabdominelle retromuskuläre umbilikale Patchplastik [r-TARUP]) beschrieben, die Operationsschritte detailliert dargestellt und in den begleitenden Videos demonstriert [5]. Dieser Videobeitrag ist der zweite Teil einer Serie von drei Beiträgen über die robotische Hernienchirurgie, Teil I beschreibt die Leistenhernienversorgung [6].

## Indikationen

Die Indikationen zur endoskopisch-robotischen Reparation primär ventraler und inzisionaler Hernien sind prinzipiell ähnlich wie die zu konventionellen laparoskopischen Verfahren und richten sich auch nach dem Risikoprofil des Patienten [2, 4, 7]. Bei adipösen Patienten oder bei bekannter Rektusdiastase hat der robotische Zugang gegenüber offenen Verfahren (dem PUMP-Verfahren z. B.) den Vorteil, dass auch asymptomatische zusätzliche Befunde mitversorgt werden [1].

Die Leitlinien empfehlen bei Umbilikalhernien mit einem Durchmesser größer als 1 cm die Netzimplantation [7]. Symptomatische Umbilikalhernien bei adipösen Patienten, bei Patienten mit großem intraabdominellem Druck sowie epigastrische Hernien sind – wie auch inzisionale Hernien mit einem Durchmesser von bis zu 7 cm – eine gute Indikation für den robotischen Eingriff. Der Ätiologie gemäß werden primär ventrale Hernien (<4 cm) mit Netzimplantation im präperitonealen Raum versorg (z. B. rv-TAPP), da kaum narbige Verwachsungen des Peritoneums am Bruchring bestehen. Primär ventrale Hernien mit einem Durchmesser >4 cm und Inzisionalhernien (<7 cm Durchmesser) werden hingegen eher mit Netzimplantation im Retrorektusraum (z. B. r‑Rives bzw. r‑TARUP) versorgt. Bei Hernien mit einer Breite von über 8 cm ist aus Perspektive der Robotik die Ausweitung des Eingriffes auf den „transversus abdominis release“ (r-TAR) sinnvoll (siehe hierzu Teil III dieser Serie in *Der Chirurg*, in Vorbereitung).

## Patientenaufklärung

Das minimal-invasive Vorgehen und die Anwendung des Operationsroboters werden dargestellt. Auf postoperative Komplikationen im Allgemeinen wie postlaparoskopische Schulterschmerzen, Nachblutung, Serombildung sowie das Auftreten chronischer Schmerzen oder Taubheitsgefühl der Haut wird hingewiesen. Die Lagerung auf dem Operationstisch mit Option zur Extension der Wirbelsäule wird thematisiert. Bei schlankem Körperbau kann es im Bereich der Haut über der Hernienreparation zu einer Wulstbildung kommen, welche sich mit großer Wahrscheinlichkeit im Laufe der ersten 3 bis 6 Monate postoperativ vollständig glättet.

Die Punktionsstelle der Veres-Nadel linkssubkostal und die Rasur des Abdomens und des rechten Oberschenkels (für die Neutralelektrode) werden angesprochen. Die Ausweitung des Eingriffes auf die gesamte Linea alba sowie die Erweiterung des Eingriffes von der rv-TAPP zum Retrorektusraum (r-Rives bzw. r‑TARUP) wird je nach intraoperativem Befund der Einschätzung des Operateurs überlassen. Als zu erwartende Rezidivrate werden die verfügbaren Ergebnisse der konventionellen Reparationen genannt (ca. 2–8 % auf 5 Jahre). Die Implantation eines nichtresorbierbaren, flachen, großporigen Netzes (ggf. auch in der Magnetresonanztomographie sichtbar), mit oder ohne Fixationshäkchen, wird besprochen. Die Patienten werden über kosmetische Optimierungsmöglichkeiten der postoperativen Narbenbehandlung beraten.

Zur Anwendung des Roboters erklären wir den Patienten, dass es kein eigentlicher Roboter ist, sondern ein Präzisionsinstrument, das ausschließlich von den Chirurg*innen geführt wird.

## Anästhesie und Lagerung

Am Tag der Operation, in der Tagesklinik, erfolgt ein letztes Gespräch mit dem Patienten, die Bruchlücke wird mit Filzstift auf der Haut markiert und die schriftliche Einwilligung kontrolliert. Der Zugang zur vorderen Bauchdecke erfolgt über die linke Patientenseite, der DaVinci Xi (Intuitive Surgical, USA) wird von der rechten Patientenseite angesteuert. Der Patient wird in Rückenlage linksbündig auf einer Antirutschmatte (Pink-Pad) auf den Trumpf-Operationstisch positioniert (Trumpf-Medical, Deutschland), der linke Arm wird etwas unterhalb der Tischebene angelagert; der rechte Arm wird für die Narkose ausgelagert (ipsilateral zur Position des DaVinci Xi), Gesicht und Beatmungstubus werden mit einem am Operationstisch montierten Metallrahmen geschützt. Der DaVinci Xi und der Trumpf-Tisch sind über Bluetooth gekoppelt und bewegen sich synchron. Der Eingriff wird unter Vollnarkose durchgeführt; die Relaxation muss bis zum Ende des Eingriffes bzw. bis zum Abdocken des Robotersystems optimal sein, bei Bedarf wird die neuromuskuläre Blockade am Ende des Eingriffes antagonisiert. Die Patienten bekommen eine perioperative Antibiotikaprophylaxe mit Cefuroxim 1,5 g (alternativ Clindamycin 600 mg).

Bei sehr kleinen Patienten oder bei Portpositionierung von kaudal (z. B. Zugang aus der „Bikini-Linie“, siehe unten) muss bei der Lagerung die Flexion des Operationstisches berücksichtigt werden. Die physiologische Dorsalextension der Lendenwirbelsäule (LWS) beträgt 30–35°; auf dem Trumpf-Operationstisch ist es bei Einstellung der Rückenplatte auf −20° („re-flex“) für den wachen Patienten angenehm und erträglich. Alternativ ist die Extension im Hüftgelenk hilfreich, welche physiologisch 15° beträgt; auf dem Trumpf-Tisch ist es bei Einstellung der Beinplatte auf −20° (wegen des konkomitanten Abkippens des Beckens und der Extension der LWS) für den wachen Patienten angenehm und erträglich. Beide Positionen bieten für die Roboterinstrumente ausreichend Bewegungsfreiraum von kaudal.

## Übersicht der relevanten Anatomie der vorderen Bauchdecke

Das *Peritoneum* wird durch zahlreiche von der hinteren Rektusscheide kommenden Perforansgefäßen durchblutet. Im Bereich der vorderen Bauchdecke umschließt das Peritoneum embryologisch bedingt supraumbilikal das Lig. teres hepatis (die obliterierte V. umbilicalis) mit dem Lig. falciforme und infraumbilikal das Lig. umbilicale medianum (der obliterierte Urachus) sowie beidseits die Pars occlusa der respektiven Aa. umbilicales, welche in den Plicae umbilicales laterales verlaufen. Diese Strukturen sind zwischen Peritoneum und Bauchdecke im präperitonealen Fettgewebe eingebettet und konvergieren von kranial und kaudal im Nabel, wo sie im vernarbten Ansatz der ehemaligen Nabelschnur am Nabelgrund blind enden. Je nach Körperbau erstreckt sich dieses präperitoneale Fettgewebe im Bereich des Nabels auf 1–4 cm lateral der Mittellinie; subxiphoidal ist es bis zu 15 cm breit und im Unterbauch mündet es im Spatium Retzii [8]. Die Schicht zwischen dem präperitonealen Fettgewebe und der (posterioren) Linea alba ist die Ebene für die präperitoneale Netzimplantation (Ebene „J“; [9]). Bei der Präparation des präperitonealen Raums um die umbilikale Bruchpforte herum müssen die darin endenden Ligamente durchtrennt werden, damit das Netz im Anschluss flächig (ohne Wellung) der Fascia endoabdominalis anzuliegen kommt.

In dieser Präparationsebene verlaufen auch die Gefäße, die im Erwachsenenleben den Nabel versorgen. Die *Durchblutung des Nabels* ist mehrfach durch *Kollateralen* von beiden Seiten gesichert:

- durch den subdermalen Plexus,
- die Aa. epigastricae inferiores (welche zwischen rechts und links mehrfach anastomosieren),
- durch kleine Gefäßäste entlang des Lig. teres hepatis und
- über Gefäße entlang des Lig. umbilicale medianum [10].

Studien aus dem Bereich der plastischen und rekonstruktiven Chirurgie haben gezeigt, dass das Vorhandensein einer A. epigastrica inferior auf nur einer Seite ausreichend ist, um die Durchblutung des Nabels zu gewährleisten [10]. Äste der Aa. epigastricae inferiores erreichen den Nabel vom M. rectus abdominis aus und gelangen als *Perforansgefäße* durch die vordere Rektusscheide nach subkutan, wo sie mit subkutanen Ästen der superfizialen epigastrischen Gefäße (Arterien und Venen, respektive) anastomosieren. Im Durchschnitt sind um den Nabel herum 5,3 arterielle Perforansgefäße [11]. Im infraumbilikalen Bereich ist der venöse Abfluss des Nabels über reiche polygonale Netzwerke zwischen beiden Vv. epigastricae superficiales gegeben [12].

Die kollagenen Fasern der *Rektusscheide* (Vagina musculi recti abdominis) münden medialseitig durch Dekussierung aus den Laminae anterior et posterior in der Linea alba. Die hinteren Rektusscheiden enden kaudal beidseits respektive am Ligamentum arcuatum, zwischen Ligamentum arcuatum und Symphyse sind die Mm. recti nach dorsal nur von der Fascia recti propria bedeckt. Im Rahmen der Präparation des retrorektalen Raumes (Ebene „F“; [9]) erfolgt der Übergang von der linksseitigen zur rechtsseitigen Rektusscheide durch Ablösung der respektiven Lamina posterior ca. 3 mm lateral der Dekussierungsebene; dadurch entsteht eine Verbindung zwischen dem retrorektalen Raum (Ebene „F“; [9]) und dem präperitonealen Raum (Ebene „J“; [9]); dieser Raum wird nach rechtslateral durch Ablösung der kontralateralen Lamina posterior wiederum 3 mm lateral der Linea alba zur eigentlichen TARUP- bzw. Rives-Ebene erweitert (siehe unten auch Abb. 2c/3).

## Präperitoneale Netzimplantation – Zugang von linkslateral (Onlinevideo 1/1. Teil)

**Figure 1:**
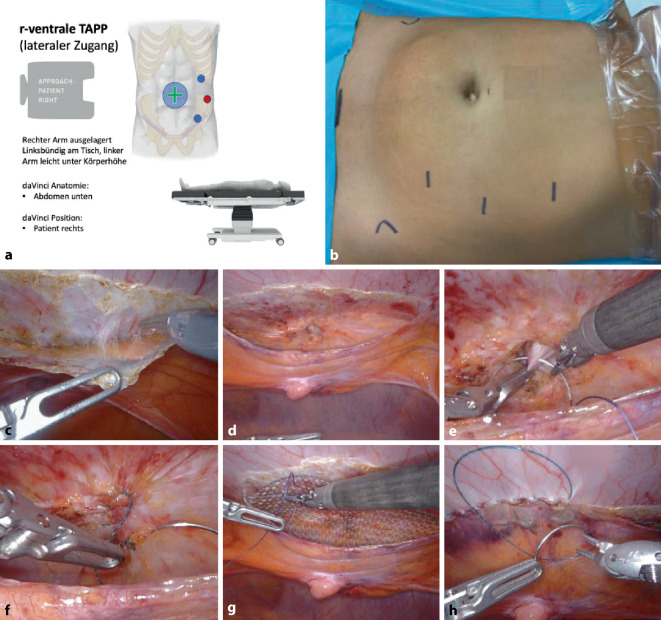

Die Ports sind auf der linken Körperseite positioniert, der DaVinci Xi steht rechts (Einstellung am DaVinci-Xi-Cardboard: Abdomen unten, Patient rechts; Abb. 1).

Begonnen wird mit dem WHO-Team-Time-out, gefolgt vom Repetieren der Operationsschritte auf der intraoperativen Checkliste (s. Zusatzmaterial online Supplement Material 1). Das Pneumoperitoneum wird über eine Veres-Nadel linkssubkostal (12 mm Hg) angelegt. Nach dem Erstellen des Pneumoperitoneums wird die Distanz zum Hernienbefund gemessen, die Ports positioniert und die anatomische Beschaffenheit der umbilikalen Plicae inspiziert. Wenn von supraumbilikal und infraumbilikal die Plicae mit üppigem präperitonealem Fettgewebe am Nabel konvergieren (bei Umbilikalhernie) oder wenn bei epigastrischer Hernie diese von ausreichend präperitonealem Fettgewebe umringt wird, ist der präperitoneale Zugang meist gut durchführbar. Wenn das Peritoneum sehr dünn ist und kaum präperitoneales Fettgewebe vorhanden ist sowie bei einem Herniendurchmesser über 4 cm besteht aus technischen Gründen die Indikation zur Erweiterung des Eingriffes auf den Retrorektusraum (siehe unten). In der folgenden Beschreibung der Technik wird die Nabelhernie als Beispiel genommen, epigastrische Hernien werden analog präpariert.

Mit dem Prograsp-Forceps-Instrument (das abgewinkelte Instrument misst 4 cm, die Länge des nichtisolierten Bereiches beträgt 4,5 cm) oder mit dem Lineal wird die laterale Präparationsgrenze abgeschätzt und mit monopolaren Koagulationspunkten auf dem Peritoneum markiert, um später eine ausreichende Netzüberlappung zu gewährleisten (Zusatzmaterial online Video 1, 01:08–01:20 min).

Der Zugang zum präperitonealen Raum wird parallel zur Linea alba und in einem lateralen Abstand von ca. 5–6 cm zu ihr über eine Länge von ca. 12 cm geschaffen (Abb. 1c). Präpariert wird zwischen Peritoneum und Fascia endoabdominalis. Beim Erreichen der Linea alba muss diese – besonders kranial des Nabels – auf konkomitante Zusatzbefunde im Sinne asymptomatischer epigastrischer Hernien exploriert werden (Zusatzmaterial online Video 1, 02:13–02:25 min).

Bevor die Umbilikalhernie und der begleitende präperitoneale Fettprolaps geborgen werden, bietet sich für eine bessere Übersicht zunächst die kaudale Präparation der Linea alba an, wobei wie oben beschrieben vor allem auch die im Nabel mündenden Ligamente durchtrennt werden. Der begleitende präperitoneale Fettkörper wird in toto geborgen und der peritoneale Bruchsack vorsichtig von der dünnen Nabelhaut abgelöst (Cave: Koagulationsschaden der Nabelhaut; Abb. 1d).

Nach Revision der Blutstillung und Messung der zu verwendenden Netzgröße, erfolgt die Reinsertion des Nabels; der technische Operationsassistent (TOA) oder der Assistent am Patienten drückt mit der Zeigefingerspitze den Nabelgrund nach innen und dieser wird mit einer Vicryl-Naht (SH-Nadel) gefasst; der/die Konsolenchirurg*in entfernt das Gesicht aus der Immersionsluke des DaVinci Xi, wodurch die Roboterarme für die Dauer der „Abwesenheit“ aus der Konsole unbeweglich bleiben (Abb. 1e); dies erlaubt dem/der Chirurg*in, die Konfiguration der neuen Nabelgrube am Patienten zu überprüfen, bevor abschließend der Nabelgrund an den Unterrand der Umbilikalpforte fixiert wird.

Wenn keine Rektusdiastase besteht bzw. die Raffung der gesamten Linea alba nicht mit dem Patienten besprochen wurde, wird die Bruchpforte mit einer transversal verlaufenden Naht mit V‑Lok 3‑0 USP (Medtronic Deutschland) verschlossen (Zusatzmaterial online Video 1, 02:26–02:44 min; Abb. 1f). Anschließend wird das flache großporige Netz (Dynamesh Endolap Visible) auf die respektive Größe zugeschnitten und eingerollt über den kranialen Port eingeführt. Es folgen die Ausrollung, Positionierung und Fixierung des Netzes mit 4 lockeren Ecknähten (resorbierbares Nahtmaterial; Zusatzmaterial online Video 1, 02:48–03:20 min; Abb. 1g). Schließlich wird das Peritoneum mit einer fortlaufenden V‑Loc-3‑0 USP-Naht von kaudal nach kranial verschlossen und der Fadenstumpf nach medial extraperitonealisiert (Abb. 1h).

Nach Zählkontrolle der Operationsmaterialien und Revision des Operationssitus wird der Eingriff beendet.

## Retrorektale Netzimplantation – Zugang von linkslateral (Onlinevideo 2)

**Figure 2:**
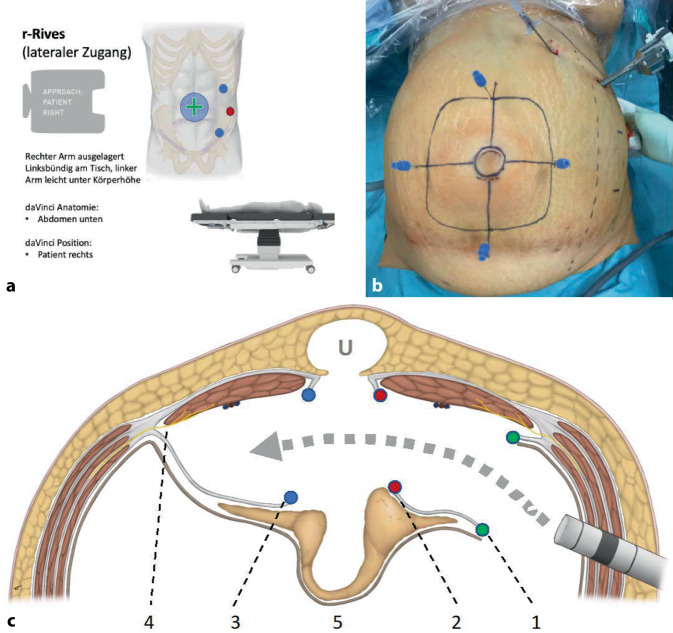

Die Ports sind auf der linken Körperseite positioniert, der DaVinci Xi steht rechts (Einstellung am DaVinci-Xi-Cardboard: Abdomen unten, Patient rechts; Abb. 2a).

Begonnen wird mit dem WHO-Team-Time-out, gefolgt vom Repetieren der Operationsschritte auf der intraoperativen Checkliste (s. Zusatzmaterial online Supplement Material 1). Das Pneumoperitoneum wird über die Veres-Nadel linkssubkostal (12 mm Hg) angelegt. Nach dem Erstellen des Pneumoperitoneums wird die Distanz zu der geplanten Portpositionen zum Hernienbefund gemessen und die laterale Begrenzung des M. rectus abdominis abgeschätzt; alternativ werden Bruchlücke und geplante Netzgröße auf die Bauchdecke eingezeichnet (Abb. 2b). Die Portpositionierung (2-mal 8 mm, 1‑mal 12 mm) wird geprüft: visuelle Kontrolle der Porttiefe im stationären Punkt, Ausschluss von Blutung oder Darmläsion und ggf. Adhäsiolyse. Zur Orientierung der inneren Weite der Präparation wird die auf die Haut eingezeichnete Netzgröße mit transparietalen Nadeln punktiert (Abb. 2b). Alternativ kann die laterale Abstandsmessung zur Hernie mit dem Prograsp Forceps (das abgewinkelte Instrument misst 4 cm) oder mit dem Lineal erfolgen, um eine ausreichende Netzüberlappung zu gewährleisten.

**Figure 3:**
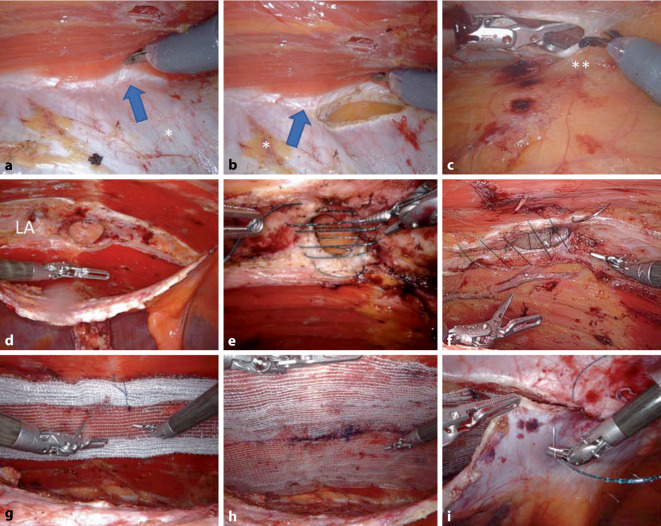

Es erfolgt der monopolare Einstieg in die laterale Begrenzung der linksseitigen hinteren Rektusscheide (Abb. 2c/1, parallel zur Linea alba und 5–8 cm lateral derselben (Zusatzmaterial online Video 2, 01:30–02:07 min). Der M. rectus abdominis wird von der hinteren Rektusscheide mit sparsamer monopolarer Präparation abgelöst, die epigastrischen Gefäße werden geschont. Der mediale Rand der linksseitigen Rektusscheide wird erreicht (Abb. 2c/2 und 3a); hier werden die zwischen der hinteren und vorderen Rektusscheide dekussierenden und die Linea-alba-bildenden Kollagenfasern identifiziert und geschont (Zusatzmaterial online Video 2, 02:23–02:43 min; Abb. 3a). Dann erfolgt der Einstieg in den präperitonealen Raum durch eine zweite (medial-)longitudinale Eröffnung der hinteren Rektusscheide (Abb. 2c/2 und 3b). Cave: Sollte ungewollt die vordere Rektusscheide eröffnet werden, gelangt man mit der Präparation fälschlicherweise in das Subkutangewebe, es wird die „Onlay“-Schicht präpariert und die Mittellinie wird mechanisch instabil.

Die Linea alba wird nun von kranial nach kaudal exploriert und die Hernienlücken freipräpariert, der Bruchsack wird parietalisiert um (gemeinsam mit dem präperitonealen Fettgewebe) als Brücke zwischen beiden hinteren Rektusscheiden zu dienen (Abb. 2c/5). Schonung der umbilikalen Haut. Beim Erreichen des rechtslateralen Randes der Linea alba erkennt man durch die dünne hintere Rektusscheide der Gegenseite den kontralateralen (rechten) M. rectus abdominis (Abb. 2c/3 und 3c); nun wird die rechte hintere Rektusscheide ebenfalls parallel zur Linea alba longitudinal eröffnet und vom M. rectus abdominis abgelöst (Zusatzmaterial online Video 2, 03:42–04:05 min). Die laterale Präparation sollte bis mindestens 5–8 cm lateral der Bruchlücken reichen, allerdings muss genauestens auf die hier verlaufenden Nerven geachtet werden, die von lateral in den M. rectus abdominis gelangen, um keine Bauchdeckenlähmung zu verursachen (Abb. 2c/4).

### Reinsertion des Nabels (bei Umbilikalhernie).

Falls eine umbilikale Hernie besteht, wird nun die Hypodermis des Nabels mit einer resorbierbaren Naht an den kaudalen Rand der Bruchlücke fixiert (Zusatzmaterial online Video 2, 04:35–05:02 min); da es sich um einen ästhetischen Operationsschritt handelt, versichert sich das Operationsteam vor dem Knüpfen des Fadens durch Inspektion des Nabels am Patienten des zu erwartenden morphologischen Ergebnisses.

### Transversale Naht (Abb. 3e; Zusatzmaterial online Video 2, 05:03 bis 05:58 min) oder longitudinale Naht (Abb. 3f) der Bruchlücke.

Bei großen umbilikalen Hernien ist die transversale Naht eine gute Option, da die Form des Abdomens postoperativ ästhetisch wiederhergestellt ist; wenn die Umbilikalpforte (ohne konkomitante Raffung der gesamten Linea alba) longitudinal vernäht wird, hat der Patient postoperativ eine Deformität des Abdomens im Sinne einer Einengung in Höhe des Nabels. Vor allem bei schlanken Patienten (mit wenig Subkutangewebe) ist es wichtig, die isolierte Bruchlücke transversal zu verschließen. Bei konkomitanter Rektusdiastase oder mehrfachen Bruchlücken sind zwei Optionen zu erwägen: Entweder die gesamte Linea alba wird longitudinal gerafft oder das Netz wird als Bridging eingelegt, um (auch wiederum bei sehr schlanken Patienten) eine Deformität der Mittellinie zu vermeiden; bei adipösen Patienten kann die Linea alba mit gutem ästhetischem Ergebnis longitudinal mit V‑Loc 0 USP (30 cm) schrittweise gerafft werden (Zusatzmaterial online Video 2, 06:00–06:50 min). Wir verwenden beim r‑TARUP ein auf die benötigte Größe zugeschnittenes Progrip-Netz (Medtronic) und berücksichtigen dabei die Empfehlungen von Tulloh und deBeaux [13]. Die Einführung, Ausrollung und Positionierung des Progrip-Netzes sind im beigefügten Video beschrieben (Abb. 3g, h; Zusatzmaterial online Video 2, 06:51–08:28 min). Wir fixieren sämtliche Netze (auch das Progrip-Netz) mit lockeren Ecknähten (resorbierbares Nahtmaterial). Vor dem Nahtverschluss der hinteren Rektusscheide wird das Pneumoperitoneum auf 8 mm Hg reduziert und die Hämostase über eine Zeit von 2–3 min kontrolliert. Der Verschluss der hinteren Rektusscheide erfolgt mit fortlaufender Naht mit zwei V‑Loc-Nähten, eine beginnend von kranial und eine beginnend von kaudal, beide Fadenstümpfe werden (nach medial hin) extraperitonealisiert (Abb. 3i). Das Peritoneum wird an der Medianlinie überprüft und ggf. entstandene Präparationslöcher mit Vicryl-Naht verschlossen. Nach Zählkontrolle der Operationsmaterialien und Revision des Operationssitus wird der Eingriff beendet. Die Durchtrittstelle des 12-mm-Ports wird transfaszial vernäht.

## rv-TAPP und r-TARUP – Zugang von kaudal/suprapubisch (Onlinevideo 1/2. Teil und 3)

**Figure 4:**
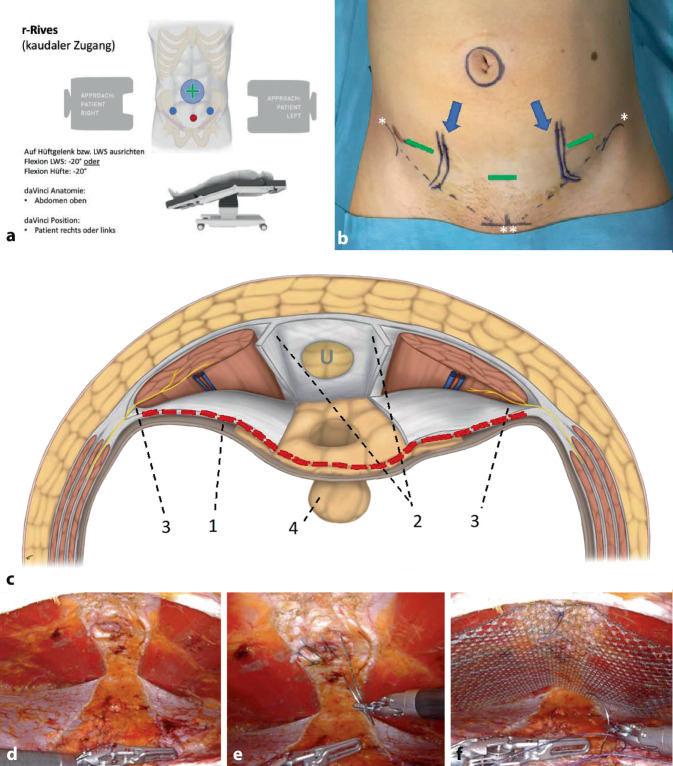

Die Ports sind suprapubisch positioniert, der DaVinci Xi steht rechts oder links. (Einstellung am DaVinci-Xi-Cardboard: Abdomen oben, Patient rechts oder links; besondere Lagerung mit Aufkippen des Patienten bzw. Hyperextension des Operationstisches; Abb. 4a).

Bei selektionierten Patienten mit umbilikaler Hernie (primär oder Rezidiv), sehr kleinem, schlankem oder athletischem Körperbau kann der Zugang von linkslateral wegen des erforderten Mindestabstands der Ports zueinander erschwert sein. In diesen Fällen oder bei ästhetischen Überlegungen zur Verbergung der Inzisionen im „Bikini-Bereich“ hat sich der kaudale Zugang bewährt. Relative, technisch bedingte Kontraindikationen sind für diesen Zugang ein sehr ausgewölbtes Abdomen und Adipositas, da die Roboterarme unter diesen Bedingungen von kaudal nicht in Position zum Erreichen der inneren Bauchdecke gebracht werden können, weil die Oberschenkel des Patienten und der anteroposteriore Durchmesser des Abdomens unter Pneumoperitoneum den Arbeitsbereich unerreichbar machen. Für den Zugang von kaudal ist auf die Lagerung zu achten (siehe oben).

Bei der Planung der Portpositionen muss auf den Verlauf der inferioren epigastrischen Gefäße und die Projektion der Inguinalnerven geachtet werden (Zusatzmaterial online Video 3. Teil, 00:43 bis 01:00 min; Abb. 4b). Ersteres wird mit dem Dopplerultraschall am steril abgedeckten Patienten oder präoperativ mit wasserfester Stiftmarkierung gemacht. Auf Höhe der Spina iliaca anterior et superior (SIAS) verlaufen die epigastrischen Gefäße posterior zum M. rectus abdominis und im Bereich seines lateralen Drittels [14]; diese Gefäßanatomie ist mit dem Dopplerultraschall exakt darstellbar; der Nervenverlauf bzw. Durchtritt durch die Bauchdecke ist von lateral kommend mediokaudal der Spina iliaca anterior et superior, wenn also die Trokare 2 cm medial der SIAS positioniert werden, passieren sie nicht die Rektusscheide, sondern die laterale Bauchdecke, mit allerdings geringem Risiko der Nervenläsion. Begonnen wird mit der Positionierung einer der lateralen Ports, dann erst der mediansuprapubische, um eine Läsion der Harnblase zu vermeiden; da hier mediansuprapubisch das präperitoneale Bindegewebe sehr elastisch ist, kann die Einführung des Trokars technisch erschwert sein (Gegendruck von innen mit einer laparoskopischen Klemme ist hilfreich).

Der präperitoneale Zugang von kaudal (rv-TAPP) ist – bis auf den Einstieg – analog zu dem oben beschriebenen (Zusatzmaterial online Video 1/2. Teil, 04:46–05:00 min; Abb. 4c–f). Der Zugang zum retrorektalen Raum (r-Rives) erfolgt über eine transversale Inzision beider hinterer Rektusscheiden (Zusatzmaterial online Video 3, 01:19–02:00 min; Abb. 4c/1, rote gestrichelte Line), mit Schaffung der Verbindung beider Räume durch longitudinale Inzision der medialen Insertion beider hinterer Rektusscheiden (Zusatzmaterial online Video 3, 02:02–02:38 min; Abb. 4c/2). Die Linea alba bleibt intakt. Die Präparation kann von hier aus bei Bedarf bis zum Xiphoid ausgedehnt werden. Die Behandlung der Bruchlücke(n), der Nabelhaut und die Prinzipien der Nabelreinsertion bzw. Nahtverschluss der Bruchlücke(n) mit oder ohne mediane Raffung der Linea alba sowie Netzpositionierung und -fixation sind die gleichen wie oben für den lateralen Zugang beschrieben. Eine transversale fortlaufende Naht beider eröffneter Rektusscheiden bzw. des Peritoneums ist der letzte Reparationsschritt.

## Kasuistik und Studiendesign

Dieser Videobeitrag fasst die Erfahrungen von 118 Operationen zusammen, welche in einem Zeitraum von Juni 2018 bis Dezember 2020 durchgeführt wurden. Es handelt sich um eine vergleichende Kohortenstudie mit 2 Operationsverfahren, wobei die Wahl des Operationsverfahrens in Abhängigkeit des intraoperativen Hernienbefundes erfolgte. Die Datenerfassung begann mit dem ersten Eingriff der Implementierungsphase des Robotikprogramms der Viszeralchirurgie am Kantonsspital Olten und beinhaltet somit auch die Zeit der Lernkurve im Umgang mit dem Operationsroboter. Die Studie wurde von der zuständigen Ethikkommission der Nordwestschweiz bewilligt (Ref. Nr. 2019-02046). Entscheidungen über Interventionen auf Ebene der Bruchpforten (Arte der Naht der Bruchlücke, Refixation des Nabels und Exploration der gesamten Linea alba) richteten sich nach den jeweiligen Befunden. Die Patienten wurden 6 Wochen postoperativ klinisch und bei Bedarf auch sonographisch nachkontrolliert. Sämtliche Daten wurden pseudonymisiert in einer klinikinternen Datenbank erfasst, die passwortgeschützt den Untersuchern zugänglich ist. In der Regel blieben die Patienten eine Nacht stationär.

Um die Verteilung kategorialer Variablen zu vergleichen wurden je nach Stichprobenumfang der χ^2^-Test oder der exakte Fischer-Test verwendet; der *t*-Test wurde für kontinuierliche Variablen verwendet. Ein *p*-Wert unter 0,05 wurde als signifikant gewertet.

## Ergebnisse

Es wurden 88 Patienten mit präperitonealer Netzimplantation (rv-TAPP) und 30 mit Retrorektusnetzimplantation (r-Rives bzw. R‑TARUP) versorgt. Patienten in der r‑Rives-Gruppe waren signifikant älter (*p* = 0,001), es gab zwischen beiden Gruppen keine weiteren demographischen Unterschiede, weder nach Art der Tätigkeit, den Komorbiditäten noch bei der ASA(American Society of Anesthesiology)-Klassifikation (Tab. 1).

**Table 1:** 

	rv-TAPP (*n* = 88)		r‑Rives (*n* = 30)		*p*-Wert
**Alter, MW (SA)**	52,3	(±13,7)	62,1	(±13,3)	**0,001**
**Weibliches Geschlecht [*n*** **(%)]**	25	(28,4)	15	(50,0)	**0,031**
**BMI kg/m**^**2**^** [MW (SA)]**	30,7	(±6,4)	29,2	(±5,4)	0,250
**Raucher [*n*** **(%)]**	37	(42,0)	14	(46,7)	0,659
**Ethnie [*n*** **(%)]**					
*Nordeuropa*	70	(79,5)	24	(80,0)	0,957
*Mediterran*	18	(20,5)	6	(20,0)	
**Art der beruflichen Tätigkeit [*n*** **(%)]**					
*Schreibtisch*	20	(22,7)	3	(10,0)	0,062
*Körperlich anstrengend*	20	(22,7)	4	(13,3)	
*Keine Arbeit oder berentet*	16	(18,2)	12	(40,0)	
*Nicht angegeben*	32	(36,4)	11	(36,7)	
**Komorbiditäten [*n*** **(%)]**					
*Arterielle Hypertonie*	43	(48,9)	12	(40,0)	0,400
*Koronare Herzkrankheit*	10	(11,4)	3	(10,0)	0,836
*Diabetes mellitus*	13	(14,8)	7	(23,3)	0,280
*COPD*	8	(9,1)	5	(16,7)	0,252
*Thrombembolische Ereignis in Anamnese*	3	(3,4)	0	(0,0)	0,591
Tiefe Venenthrombose	3	(3,4)	0	(0,0)	0,305
Lungenembolie	1	(1,1)	0	(0,0)	0,557
*Immunsuppressive Therapie*	2	(2,3)	0	(0,0)	0,405
*Orale Antikoagulation*	16	(18,2)	6	(20,0)	0,825
DOAC	5	(5,7)	1	(3,3)	0,805
Marcumar	1	(1,1)	0	(0,0)	0,557
Plättchenaggregationshemmer	11	(12,5)	5	(16,7)	0,643
**ASA-Score**					
*ASA I*	8	(9,1)	4	(13,3)	0,425
*ASA II*	64	(72,7)	18	(60,0)	
*ASA III*	16	(18,2)	8	(26,7)	

*ASA* American Society of Anesthesiology Score, *BMI* Body-Mass-Index, *COPD *„chronic obstructive pulmonary disease“, *DOAC* „duale orale Antikoagulation“*MW* Mittelwert, *r‑Rives* robotische transabdominelle retromuskuläre umbilikale Patchplastik [r-TARUP], *rv-TAPP* robotische ventrale transabdominelle präperitoneale Patchplastik, *SA* Standardabweichung

Beide Gruppen unterschieden sich nach Art der Hernie; primär ventrale Hernien wurden häufiger als rv-TAPP, Inzisionalhernien als r‑Rives versorgt (Tab. 2). Bei 4 Patienten, bei denen eine rv-TAPP geplant war, wurde wegen zu dünnen Peritoneums ein r‑Rives durchgeführt; in einem Fall wurde bei rv-TAPP ein 4 × 4 cm großer peritonealer Einriss mit einem Stück Vicryl-Netz verschlossen; bei keinem der Patienten musste auf ein r‑IPOM (robotisch-intraperitoneales Onlay-Mesh) ausgewichen werden. Bei 48 der insgesamt 118 Patienten wurde im Bereich der Linea alba intraoperativ ein zusätzlicher Befund präpariert, der asymptomatisch war (37,5 %). Die Bruchlücken und die respektiven Defektflächen waren in der r‑Rives-Gruppe signifikant größer (*p* < 0,001); analog waren auch die Netze in der r‑Rives-Gruppe signifikant größer (Tab. 2). Die Ratio der Netzfläche zur Bruchlückenfläche war in beiden Gruppen vergleichbar (*p* = 0,142; Tab. 2). Eine Netzfixation erfolgte bei 93 % der Patienten beider Gruppen, eine subkutane Drainage wurde in nur 2 Fällen bei sehr großer umbilikaler Hernie verwendet (Tab. 2). Die rv-TAPP war mit durchschnittlich 82 min Operationszeit (Schnitt-Naht-Zeit, inklusive Andocken) signifikant kürzer, als der r‑Rives, mit durchschnittlich 109 min (Schnitt-Naht-Zeit, inklusive Andocken; Tab. 2). Der Zeitablauf von der Punktion der Veres-Nadel (Schnitt) bis zum Beginn an der Konsole betrug 7–9 min.

**Table 2:** 

	rv-TAPP (*n* = 88)		r‑Rives (*n* = 30)		*p*-Wert
**Art der Hernie [*n*** **(%)]**					
*Primär umbilikal*	53	(60,2)	10	(33,3)	**<0,001**
*Primär epigastrisch*	22	(25,0)	1	(3,3)	
*Primär lateral/Spieghel*	3	(3,4)	0	(0,0)	
*Inzisional umbilikal (EHS M3)*	4	(4,5)	2	(6,7)	
*Inzisional (andere; EHS M2-M3-M4)*	6	(6,8)	17	(56,7)	
**Weitere Bruchlücken der Linea alba [*n*** **(%)]**	38	(43,2)	10	(33,3)	0,797
**Größe der Bruchlücke [MW (SA)]**					
*Länge in cm*	2,3	(±1,1)	4,9	(±1,1)	**<0,001**
*Breite in cm*	2,2	(±1,0)	4,2	(±1,0)	**<0,001**
*Fläche Defekt in cm*^*2*^	8,8	(±9,4)	20,1	(±17,7)	**<0,001**
**Bruchlückenverschluss [*n*** **(%)]**					
*Keiner*	13	(14,8)	3	(10,0)	**<0,001**
*Mediane Naht*	1	(1,1)	8	(26,7)	
*Transversale Naht*	74	(84,1)	19	(63,3)	
**Netzgröße [MW (SA)]**					
*Länge in cm*	11,6	(±3,5)	16,1	(±4,0)	**<0,001**
*Breite in cm*	9,0	(±2,1)	12,7	(±2,9)	**<0,001**
*Fläche in cm*^*2*^	107,8	(±56,0)	205,5	(±77,6)	**<0,001**
**Ratio Netzfläche:Bruchlückenfläche** [**MW (SA)]**	30,1	(±50,1)	16,5	(±12,8)	0,142
**Netzart [*n*** **(%)]**					
*Dynamesh Endolap Visible*	73	(83,0)	2	(6,7)	**<0,001**
*Progrip*	15	(17,0)	27	(90,0)	
*Symbotex*	0	(0,0)	1	(3,3)	
**Netzfixierung [*n*** **(%)]**					
*Keine*	1	(1,1)	0	(0,0)	0,828
*Vicryl-Naht*	82	(93,2)	28	(93,3)	
*V‑Loc-Naht*	5	(5,7)	2	(6,7)	
**Subkutane Drainage [*n*** **(%)]**	2	(2,3)	0	(0,0)	0,405
**Schnitt-Naht-Zeit in min [MW (SA)]**	82,9	(±21,0)	109,1	(±32,4)	**<0,001**

*EHS* Klassifikation der Europäischen Herniengesellschaft [24], *MW* Mittelwert, *r‑Rives* robotische transabdominelle retromuskuläre umbilikale Patchplastik [r-TARUP], *rv-TAPP* robotische ventrale transabdominelle präperitoneale Patchplastik, *SA* Standardabweichung

Der Krankenhausaufenthalt war in der rv-TAPP-Gruppe kürzer als in der r‑Rives-Gruppe (1,5 vs. 2,7 Tage, respektive; *p* < 0,001; Tab. 3). Es gab keinen Unterschied in der Inzidenz von Serom, Hämatom oder Hautnekrose; insgesamt gab es keinen Unterschied beim Auftreten unerwünschter Ereignisse zwischen beiden Gruppen; der einzige Unterschied war eine signifikante Häufung von Seromen Typ II in der r‑Rives-Gruppe (*p* < 0,001), die Gesamtzahl der Serome war allerdings vergleichbar (Tab. 3).

**Table 3:** 

	rv-TAPP (*n* = 88)		r‑Rives (*n* = 30)		*p*-Wert
**Ambulanter Eingriff [*n*** **(%)]**	15	(17,0)	3	(10,0)	0,354
**Krankenhausaufenthaltsdauer in Tagen [MW (SA)]**	1,5	(±0,6)	2,7	(±1,7)	**<0,001**
**VAS am 1. postoperativen Tag [MW (SA)]**^**a**^	2,3	(±2,0)	2,6	(±1,5)	0,529
**Unerwünschte Ereignisse [*n* (%)]**					
*Wundereignisse (SSO) *	16	(18,2)	9	(30,0)	0,171
Serom (nach Morales-Conde)	14	(15,9)	7	(23,3)	0,358
– Grad I	1	(1,1)	–	–	**<0,001**
– Grad II	11	(12,5)	5	(16,7)	
– Grad III	2	(2,3)	0	(0,0)	
– Grad IV	–	–	2	(6,7)	
Hämatom	3	(3,4)	3	(10,0)	0,155
Hautnekrose	–	–	1	(3,3)	0,085
*Ungeplante Wiedervorstellung bei Schmerzen*	5	(5,7)	1	(3,3)	0,613
*Verzögertes Einsetzen der Darmpassage *	1	(1,1)	1	(3,3)	0,557
*Lungenembolie *	2	(2,3)	–	–	0,405
*Clavien-Dindo [n (Patienten)]*					
Grad I	23	(20)	9	(8)	0,661
Grad II	2	(2)	–	–	0,405
Grad III	–	–	1	(1)	0,085
Grad IV	–	–	1	(1)	0,085
*CCI [MW (SA)]*	2,7	(±5,6)	4,4	(±8,1)	0,191
**Klinische Kontrolle nach 6 Wochen [*n* (%)]**					
*Erfolgt *	74	(84,1)	28	(93,3)	0,201
*Rezidiv*	–	–	–	–	1,000
*Bauchdeckenschmerzen*	5	(6,7)	–	–	0,161
*Serom*	10	(13,3)	7	(25,0)	0,155
*Hämatom*	1	(1,3)	1	(3,7)	0,446

*CCI* Charlson Comorbidity Score, *r‑Rives* robotische transabdominelle retromuskuläre umbilikale Patchplastik [r-TARUP], *rv-TAPP* robotische ventrale transabdominelle präperitoneale Patchplastik, *SSO* „surgical site occurrence“, *VAS* visuelle Analogskala

^a^Bei Patienten mit stationärem Aufenthalt

Bei der näheren Analyse der Wundkomplikationen („surgical site occurrence“, SSO), mit Gegenüberstellung von SSO^+^ bzw. SSO^–^, waren beide Verfahren vergleichbar. Auffallend ist, dass in dieser Subgruppenanalyse Alter, BMI und Nikotinkonsum keinen negativen Einfluss auf das Ergebnis hatten. Auch die Ratio der Netzfläche zur Bruchlückenfläche korrelierte nicht mit SSOs (Tab. 4).

**Table 4:** 

	SSO^−^		SSO^+^		*p*-Wert
	(*n* = 93)		(*n* = 25)		
**Alter [MW (SA)]**	54,1	(±14,2)	57,6	(±14,1)	0,265
**Weibliches Geschlecht [*n*** **(%)]**	29	(31,2)	11	(44,0)	0,229
**BMI kg/m**^**2**^** [MW (SA)]**	29,9	(±6,2)	31,8	(±5,8)	0,179
**Raucher [*n*** **(%)]**	38	(40,9)	13	(52,0)	0,318
**Komorbiditäten [*n*** **(%)]**					
*Arterielle Hypertonie*	42	(45,2)	13	(52,0)	0,542
*Koronare Herzkrankheit*	8	(8,6)	5	(20,0)	0,106
*Diabetes mellitus*	14	(15,1)	6	(24,0)	0,289
*COPD*	11	(11,8)	2	(8,0)	0,587
*Orale Antikoagulation*	18	(19,4)	4	(16,0)	0,702
*ASA-Score*					
ASA I	11	(11,8)	1	(4,0)	0,347
ASA II	65	(69,9)	17	(68,0)	
ASA III	17	(18,3)	7	(28,0)	
**Hernienart [*n*** **(%)]**					
*Umbilikal*	50	(53,8)	13	(52,0)	0,764
*Epigastrisch*	17	(18,3)	6	(24,0)	
*Inzisional*	23	(24,7)	6	(24,0)	
*Spieghel*	3	(3,2)	–	–	
**Zweiter Hernienbefund [*n*** **(%)]**	34	(36,6)	13	(52,0)	0,567
**Bruchlücke, Defektfläche in cm**^**2**^ [**MW (SA)]**	10,6	(±11,5)	15,7	(±17,2)	0,085
**Prozedur [*n*** **(%)]**					
*rv-TAPP*	72	(77,4)	16	(64,0)	0,171
*r‑Rives*	21	(22,6)	9	(36,0)	
**Bruchlückenverschluss [*n*** **(%)]**	19	(20,4)	8	(32,0)	0,221
**Größe des Netzes in cm**^**2**^ [**MW (SA)]**	132,9	(±77,8)	131,9	(±65,8)	0,953
**Ratio Netzfläche:Bruchlückenfläche **[**MW (SA)]**	29,7	(±48,7)	14,9	(±10,3)	0,143
**Netzart [*n*** **(%)]**					
*Dynamesh*	61	(65,6)	14	(56,0)	0,553
*Progrip*	31	(33,3)	11	(44,0)	
*Symbotex*	1	(1,1)	–	–	
**Schnitt-Naht-Zeit**^**a**^ in** min [MW (SA)]**	88,9	(±26,8)	91,9	(±27,3)	0,625

*ASA* American Society of Anesthesiology Score, *COPD *„chronic obstructive pulmonary disease“,*r‑Rives* robotische transabdominelle retromuskuläre umbilikale Patchplastik [r-TARUP], *rv-TAPP* robotische ventrale transabdominelle präperitoneale Patchplastik, *SSE* „surgical site event“ (Wundkomplikation), *SSO* „surgical site occurrence“

^a^Schnitt-Naht-Zeit beinhaltet die gesamte Operationszeit, inklusive des An- und Andockens

## Diskussion

Die ideale Reparation abdomineller Hernien der Medianlinie ist noch nicht etabliert. Daten der vergangenen Jahrzehnte haben allerdings einen guten Überblick über Vorteile und Grenzen einzelner Verfahren geliefert:

- BMI, Alter und Nikotinkonsum sind die bedeutendsten Risikofaktoren für Komplikationen;
- Bruchpfortengröße und Netzüberlappung korrelieren mit Rezidiv;
- offene Verfahren haben mehr Komplikationen und weniger Rezidive, minimal-invasive Verfahren weniger Komplikationen und mehr Rezidive;
- Hämatome und Serome kommen bei den unterschiedlichen Verfahren ähnlich häufig vor [2, 4, 13, 15–17].

Eine aktuelle Delphi-Studie hat die Schichten der Bauchdecke, welche für die Netzreparationen verfügbar sind, neu zusammengefasst und die vielfältigen Möglichkeiten der Netzpositionen verdeutlicht [9]. Während in den vergangenen beiden Jahrzehnten laparoskopische Reparationen meist als IPOM-Technik mit oder ohne Bruchlückenverschluss durchgeführt wurden (mit Netzen mit Kontakt zu den Abdominalorganen), besteht aktuell die Tendenz, minimal-invasiv Netze aus der Bauchhöhle in eine der verschiedenen verfügbaren Schichten zu verlegen. Ein Pionier dieser Idee war Marc Miserez der Universität Leuven in Belgien, der bereits 2002 ein endoskopisch totalextraperitoneales Verfahren (nach heutiger Definition eine Retrorektusreparation) für die Ventralhernie an 15 Patienten beschrieben hat [18]. Später wurde auch der transabdominelle Zugang zum Retrorektusraum beschrieben [19]. Der Benchmark, an dem sich jede Reparation von Umbilikalhernien messen muss, ist wahrscheinlich die E/MILOS-Technik, bei der in einer Kohortenstudie an 520 Umbilikalhernien fast ideale Ergebnisse von 1,2 % Komplikationen, 0,0 % Infektionen und 0,0 % Rezidiven nach einem Jahr beschrieben wurden; behandlungsbedürftige chronische Schmerzen traten bei 0,6 % der Patienten auf [3]. Ob allerdings jede Umbilikalhernie ein so großes Netz braucht, wie bei E/MILOS üblich, bleibt zu diskutieren, nicht zuletzt, weil gerade jüngere Patienten mit einer erneuten abdominellen Operation im Laufe ihres Lebens rechnen müssen, und Netze größer als nötig problematisch für den Zugang zum Abdomen werden können. Die Daten zum E/MILOS müssen in Zukunft noch von anderen Zentren extern validiert und in randomisiert-kontrollierten Studien überprüft werden.

Konsensfähig scheint aktuell die Erkenntnis, dass moderne Verfahren die Vorteile der offenen Reparation (morphologische und funktionelle Reparation, extraperitoneales Netz, geringe Rezidivrate) mit denen der minimal-invasiven Verfahren (weniger Komplikationen) vereinen müssen [20]. Das konventionell-laparoskopische LIRA(„laparoscopic line alba stapler repair“)-Verfahren ist ein Schritt in diese Richtung, die Bruchlücke wird auf Faszienebene verschlossen, das Netz wird allerdings nach wie vor in IPOM-Position implantiert [21].

Mit der Robotik ist das Ziel der Extraperitonealisierung der Netze mit morphologischer Rekonstruktion und minimal-invasivem Verfahren einer neuen Qualität näher gekommen. Nadia Henriksen aus Kopenhagen in Dänemark hat in einer Metaanalyse gezeigt, dass die Robotik bei ventralen Hernien von Vorteil sein kann [22]. In der aktuellen Kohortenstudie werden zwei robotische Verfahren untersucht: die transabdominelle präperitoneale Netzimplantation (rv-TAPP) und die transabdominelle Retrorektusnetzimplantation (r-Rives bzw. r‑TARUP). Kudsi et al. haben gezeigt, dass die rv-TAPP eine Lernkurve von ca. 46 Patienten hat, bis die Ablösung des Peritoneums technisch optimiert ist, mit Reduzierung der Peritonealeinrisse von 63 % auf 11 %; die durchschnittliche Schnitt-Naht-Zeit betrug bei 105 Patienten 54 min [23]. In unserer Studie war die Schnitt-Naht-Zeit mit 82 min länger, obwohl die Größe der Bruchlücken vergleichbar ist (Tab. 2). Wahrscheinlich ist dies dadurch bedingt, dass in unserer Studie jeder Eingriff mit der doppelten Konsole ein Weiterbildungseingriff war; allerdings zeigen unsere Daten, dass auch unter Weiterbildungsbedingungen eine stabile Operationsdauer von deutlich unter 90 min eingehalten werden kann. Im Unterschied zur Studie von Kudsi et al. ist die Ratio der Netzfläche zur Bruchlückenfläche in unserer Studie mit einem Mittelwert von 30,1 höher als bei jenem (19,2), was auch zur längeren Operationsdauer beigetragen haben mag (Tab. 2; [23]). Der präperitoneale Zugang ist der am wenigsten traumatisierende für die Bauchdecke; nachteilig ist, dass die breitflächige Ablösung des teils dünnen, aber sehr gut durchbluteten Peritoneums den Eingriff bei Netzen über 10 cm Breite erschweren kann, sodass bei Hernien über 4 cm Durchmesser der Retrorektusraum zu bevorzugen ist.

Die Retrorektusreparation (r-Rives bzw. r‑TARUP) führen wir in Analogie zur von Muysoms beschriebenen Technik durch [24]. Ähnlich wie in der Kohorte von Muysoms sind auch in unserer Kohorte die meisten Hernien umbilikaler Topographie, wobei bei Muysoms 7 von 42 inzisional waren, während in unserer Kohorte 20 von 30 inzisional waren (Tab. 2; [24]). Von Bedeutung ist, dass in unserer Kohorte bei den als r‑Rives versorgten Patienten mit Hernienflächen um die 20 cm^2^ eine Netzfläche-zu-Bruchflächen-Ratio von durchschnittlich 16,5 eingehalten wurde, was die theoretischen Anforderungen von Tulloh und deBeaux erfüllt (Tab. 2; [13]). Die postoperativen Komplikationen sind gering (Tab. 3).

Die Anzahl von Bruchlücken der Linea alba, die asymptomatisch sind, aber intraoperativ als Zusatzbefunde gesehen werden, ist in der Literatur nicht beschrieben, weder in den klassischen Lehrbüchern noch in einer aktuellen PubMed-Recherche. Die hier beschriebenen 37,5 % sind erstmalig publiziert und zeigen die Sinnhaftigkeit der Exploration der Linea alba in der Umgebung des symptomatischen Hernienbefundes, was bei offenen periumbilikalen Zugängen nicht möglich ist.

Der robotische Operationszugang von kaudal (oben für den präperitonealen wie für den Retrorektusraum beschrieben) ist für Hernien des Nabels und der supraumbilikalen Linea alba aus zwei Gründen sinnvoll: a) Zusatzbefunde im Sinne primär ventraler Hernien kommen infraumbilikal kaum vor (hypogastrische Hernien sind nicht beschrieben), sodass vom kaudalen Zugang aus die ausreichende distale Überlappung des Netzes über den Hauptbefund hinaus möglich ist [25]; b) auch wenn eine longitudinale Raffung der Linea alba (bei konkomitanter Rektusdiastase) geplant ist, ist die Naht im infraumbilikalen Bereich selten bis zur Symphyse nötig, da infraumbilikal die (physiologische) Diastase praktisch nicht vorkommt. Erklärt wird dies von Ranney, der postuliert, dass der obere Bereich der Linea alba dynamisch auf Druckveränderungen reagiert (Atmung und Nahrungsaufnahme), während der infraumbilikale Bereich der Linea alba ausschließlich der Stabilisierung des Gewichtes der intraabdominalen Organe dient und somit deutlich weniger dehnbar als der obere Bereich der Linea alba ist [25]. Wir haben den kaudalen Zugang bislang bei 6 Patienten durchgeführt.

Die klassischen Risikofaktoren für Komplikationen (Alter, BMI, Nikotin, Komorbiditäten) korrelierten in dieser Kohorte nicht mit mehr Komplikationen; dies kann bedeuten, dass die Robotik besonders bei Risikopatienten von Bedeutung sein kann, bzw. es würde sich lohnen, hier mehr Daten zu erheben. Allein bei der Bruchlückenfläche zeichnet sich bei größeren Hernien eine Tendenz für mehr Wundkomplikationen ab, ohne statistische Relevanz (Tab. 4). Bei morbider Adipositas kann es sinnvoll sein, vor der Versorgung der Hernie einen bariatrischen Eingriff zu machen [17, 26].

Für Hernien mit einem Durchmesser über 8 cm sind die hier beschriebenen Verfahren nicht indiziert, da keine ausreichende Netzunterfütterung möglich ist. Größere Hernien werden robotisch als „transversus abdominis release“ versorgt (r-TAR), ein Verfahren das ursprünglich von Alfredo Carbonell für offene Operationen als posteriore Komponentenseparation beschrieben wurde und im 3. Beitrag dieser Serie in *Der Chirurg* für die robotische Anwendung beschrieben wird [27].

Zuletzt noch ein Wort zur Frage der Kosten: Der r‑Rives bzw. r‑TARUP kostet 1330 CHF (davon 950 CHF für das DaVinci-Material unter Extended Use Program und 380 CHF für das Netz); im Vergleich dazu, verursacht das laparoskopische IPOM bei vergleichender Herniengröße Kosten von 2380 CHF (davon 1380 CHF für das Netz, 720 CHF für 2 Tackergeräte und 280 CHF für die Einwegtrokare); das laparoskopische IPOM verursach in unserem Hause 950 CHF höhere Materialkosten als der robotische Eingriff (der Wechselkurs zum Euro ist mit ca. 1:1 übertragbar). Wird die Umlage der Roboterwartungspauschale von 420 CHF (bei 300 Eingriffen/Jahr) je Patienten berücksichtigt, erreicht der robotische Eingriff innerhalb der DRG(„diagnosis related groups“)-Vergütung Einsparungen von 630 CHF pro Fall, im Vergleich zum laparoskopischen IPOM. Weniger postoperative Schmerzen (keine Tacker und keine transfaszialen Nähte) sowie kürzerer Spitalaufenthalt sind hier nicht berücksichtigt.

Zusammenfassend ermöglicht die robotische Technologie sichere und neue minimal-invasive Zugänge zu den verschiedenen Schichten der Bauchdecke und in den allermeisten Fällen die Extraperitonealisierung der Netze mit einer geringen Komplikationsrate. Diese Entwicklung ist die natürliche Folge der Erkenntnisse von 30 Jahren Laparoskopie und der Anfang einer neuen Zukunft.

## Fazit für die Praxis

Bei ca. jedem 3. Patienten mit primär ventraler Hernie findet sich zusätzlich zum Hauptbefund eine zweite, konkomitante, asymptomatische Hernie der Linea alba.

Die robotische Reparation ventraler und inzisionaler Hernien ...

- hat alle Vorteile minimal-invasiver Verfahren (geringe Komplikationsrate),
- integriert Vorteile der offenen Verfahren (morphologische Rekonstruktion),
- ermöglicht eine konsequente Extraperitonealisierung der Netze,
- ist ein sehr flexibles Instrument für „tailored approach“: umbilikale und epigastrische Hernien (<4 cm) werden als rv-TAPP (robotische ventrale transabdominelle präperitoneale Patchplastik) versorgt; inzisionale Hernien, große Bruchlücken (4–7 cm) sowie bei geplanter Raffung der Linea alba wird der r‑Rives bzw. r‑TARUP (robotische transabdominelle retromuskuläre umbilikale Patchplastik) durchgeführt,
- erlaubt individuelle Portpositionierung, je nach Art und Lokalisation der Hernie,
- ist ein geeigneter Eingriff für die Weiterbildung,
- ist kostengünstiger als das konventionelle laparoskopische intraperitoneale Onlay-Mesh (IPOM).

## Supplementary Information







