# Erratum zu: Robotische Hernienchirurgie II: Robotische primär ventrale und inzisionale Hernienversorgung (rv-TAPP und r-Rives/r-TARUP). Videobeitrag und Ergebnisse einer Kohortenstudie an 118 Patienten

**DOI:** 10.1007/s00104-022-01572-4

**Published:** 2022-01-17

**Authors:** Johannes Baur, Michaela Ramser, Nicola Keller, Filip Muysoms, Jörg Dörfer, Armin Wiegering, Lukas Eisner, Ulrich A. Dietz

**Affiliations:** 1 Klinik für Viszeral‑, Gefäss- und Thoraxchirurgie, Kantonsspital Olten, Baslerstrasse 150, 4600 Olten, Schweiz; 2 Klinik für Allgemein‑, Viszeral- und Gefässchirurgie, Kantonsspital Baden, Im Engel 1, 5404 Baden, Schweiz; 3 Department of Surgery, AZ Maria Middelares, Buitenring Sint-Denijs 30, 9000 Gent, Belgien; 4 Klinik und Poliklinik für Allgemein‑, Viszeral‑, Transplantations‑, Gefäß- und Kinderchirurgie, Universitätsklinikum Würzburg, Oberdürrbacher Straße 6, 97080 Würzburg, Deutschland

**Erratum zu:**

**Chirurg 2021**

10.1007/s00104-021-01450-5

Dieser Beitrag zum Thema „Robotische Hernienchirurgie. Rv-TAPP und r‑Rives/r-TARUP“ ist der zweite Teil einer Reihe von mehreren Beiträgen zum Thema. Titel und Untertitel wurden angepasst. Die Originalversion dieses Beitrags wurde korrigiert.

