# Supplementary material for: Robotic hernia repair II. English version: Robotic primary ventral and incisional hernia repair (rv-TAPP and r‑Rives or r‑TARUP). Video report and results of a series of 118 patients
Source: Chirurg. 2021 Jul 13;92(9):809–21. [Article in German] doi: 10.1007/s00104-021-01450-5 (PMC8384833; doi:10.1007/s00104-021-01450-5)
Supplement: Supplementary file 4 [file 104_2021_1450_MOESM4_ESM.pdf]

Patientenaufkleber

Datum: _____/_____/_____

Operateur: _____________

# r-ventrale TAPP
# r-Rives (TARUP)

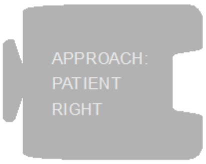


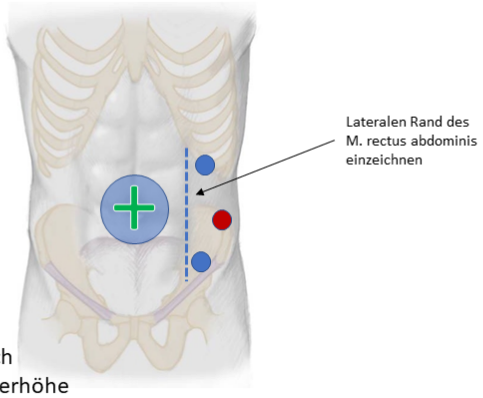


Rechter Arm ausgelagert
Körper Linksbündig zum Tisch
Linker Arm leicht unter Körperhöhe

daVinci Anatomie:
- Abdomen unten

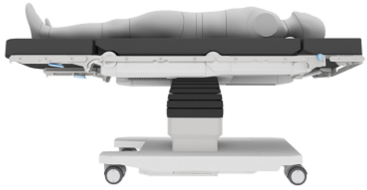

daVinci Position:
- Patient rechts

## Perioperative Prozeduren/daVinci-Checkliste (Achtung: Ersetzt nicht das WHO Team-Time-Out)

Ich habe den Aufklärungsbogen heute gesichtet und auf Inhalt und Bemerkungen hin kontrolliert: □ Ja □ Nein
Perioperative Antibiose: □ Ja □ Cefuroxim 1,5g (Gewichtsadaptiert) (Alternativ: □ Clindamycin 600mg)
Hybrid-Verfahren: □ Ja □ Nein Wenn ja, Ansage an OTA's machen.

**Vor Andocken**: Abstand Trokar-Linie zur Linea alba> 15cm; Rektusränder eingezeichnet: □ Ja □ Nein
Es handelt sich um eine Inzisionalhernie oder um ein Rezidiv: □ Ja □ Nein
Bei Rezidiv, LA/LÄ vor Beginn der Operation informiert: □ Ja □ Nein
Anzahl der Voroperationen bzw. Adhäsionsvorgeschichte bekannt: □ Ja □ Nein
Ropivacain-Infiltration der Portinzisionen vor dem Schnitt erfolgt: □ Ja □ Nein
Laparoskopische Sichtkontrolle der Trokareinstichstellen: □ Ja □ Nein

**Konsole**: Komplette Adhäsiolyse der vorderen Bauchdecke erfolgt: □ Ja □ Nein
r-Rives (Synonym: r-TARUP):
Einstieg in ipsilaterale Rektusscheide, weit lateral: □ Ja □ Nein
**Cave**: Crossover am medialen Rand der Rektusscheide: erneute Inzision der posterioren Scheide, die Linea alba bleibt dabei intakt.
**Cave**: Instabilität der Linea alba durch ungewollte Eröffnung der vorderen Rektusscheide.
**Cave**: Verletzung der sehr dünnen Nabelhaut.
Mobilisation allen Fettes aus Nabel-Pforte und epigastrischen Lücken: □ Ja □ Nein
Ausschluss weiterer Hernien bis zum Xiphoid: □ Ja □ Nein
Präparation im Retzius- und Bogros-Raum: □ Ja □ Nein
Nabelhaut mit Vicryl reinseriert: □ Ja □ Nein
Transversale V-Loc-Naht der umbilikalen Bruchlücke: □ Ja □ Nein
Netz-Messung in cm; 6-fache-Fixation (lockere Vicryl-Nähte): □ Ja □ Nein
Fortlaufende Naht der hinteren Rektusscheide (V-Loc 3-0): □ Ja □ Nein
r-ventrale TAPP:
Wie r-Rives, Zugang allerdings nur durch Inzision des Peritoneums: □ Ja □ Nein
Akzidentelle Einrisse des Peritoneums wurden vernäht: □ Ja □ Nein
Alternative: Vicryl-Netz-Interposition bei grossen Peritonealdefekten: □ Ja □ Nein

| | | | |
|---|---|---|---|
| **Abschluss**: | Hybrid-Hautresektion überschüssiger Nabelhaut: | □ Ja | □ Nein |
| | Wenn 12mm Port: Fasziennaht mit Endoclose unter Sicht erfolgt: | □ Ja | □ Nein |
| | Port-Entfernung unter Sichtkontrolle auf Nachblutung: | □ Ja | □ Nein |
| | Normale postoperative Verordnung: | □ Ja | □ Nein |

**Prästationäre Vorbereitung:**
Ggf. CT-Abdomen mit transversalen und Sagittalen Schnitten (Morphologie und Verwachsungen)
Würzburg-Inzisionalhernienklassifikation
Wenn Bruchlücke mehr als 8cm breit, r-TAR oder r-IPOM erwägen.

**Nahtmaterial und Aushilfsmaterialien:**
V-Loc 180 (grün) 3-0 USP V-20 (15cm)
Vicryl 3-0 SH (20cm)
Vicryl 3-0 ohne Nadel Für Netz-Einrollung
Lineal
DynaMesh Endolap Visible 15x10cm Netz
(Ggf.: Vicryl-Netz 28x18cm)
(Ggf. Progrip Netz 30x15cm)
Markierstift

**Lagerung:**
Rückenlage
Keine Pink-Pad
Rechter Arm wird ausgelagert
Patient liegt linksbündig am OP-Tisch
Linker Arm leicht unter Körperhöhe

**Geplante OP-Zeit:** 1-3h

**Instrumente:**
30° Optik
Prograsp Forceps
Mega SutureCut Needle Driver
Hot Shears MCS
3x 8mm daVinci Trokare
(Ggf. 1x 12mm daVinci Trokar)

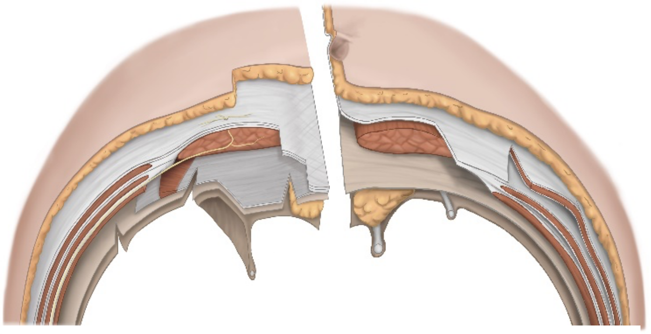

**Supraumbilikale Schichten** **Infraumbilikale Schichten**

Aus: Dietz, Fortely, Beldi, Wiegering (Hrsg.) Offene Hernienchirurgie (2012) Springer Verlag

**Hautnaht:**
Ropivacain 0,5% 10ml
Caprosyn 4-0 USP mit P-24 Nadel
Glubran-Verband

**Postoperative Verordnung:**
Mobilisation am Folgetag
Vollbelastung nach 3-14 Tagen

**CIRS-Meldung:** □ Ja □ Nein

**Swiss DRG** G09D, Zielverweildauer: **0** | 3 | 9 Tage

Geplante Entlassung: _____/_____/_____

| Ocurrence | M = morphology | S = size (cm) | RF = risk factors (+ → +++) |
|---|---|---|---|
| p (*primary*)<br>r (*recurrent*) | m (*median*)<br>u (*umbilical*)<br>sp (*suprapubic*)<br>sc (*subcostal*)<br>t (*transversal*)<br>l (*lumbal*)<br>pm (*paramedian*)<br>nc (*other*) | a x b | Obesity (BMI >25)<br>Male gender<br>Nicotine abuse<br>Wound contamination<br>Age > 45 years<br>Disease<br>2nd laparot. < 1 month<br>Emergency procedure<br>Postop. Complications<br>… |

v/i M… S…x… RF…

**Debriefing: Was kann bei der nächsten OP besser gemacht werden?**
